# Trefoil factors peptide-3 is associated with residual invasive breast carcinoma following neoadjuvant chemotherapy

**DOI:** 10.1186/s12885-019-5316-y

**Published:** 2019-02-11

**Authors:** Suhail Al-Salam, Manjusha Sudhadevi, Aktham Awwad, Mohamed Al Bashir

**Affiliations:** 1 Department of Pathology, College of Medicine & Health Sciences, United Arab Emirates University, AlAin, PO Box 17666, United Arab Emirates; 2 Department of Laboratory Medicine, Tawam Hospital, AlAin, United Arab Emirates; 3 Department of Surgery, Tawam Hospital, AlAin, United Arab Emirates

**Keywords:** Breast carcinoma, Chemoresistance, Trefoil factor-3

## Abstract

**Background:**

Breast carcinoma is the commonest cancer among UAE population and the most common cancer among females. Examination of the 5′ promoter regions of trefoil factor 3 (TFF3) gene has identified putative estrogen and progesterone receptor–DNA binding domains as direct response elements to estrogen and progesterone that are linked to breast functions or steroid regulation. The study was designed to determine the role of TFF3 in breast cancer chemoresistance with the aim of establishing TFF3 expression as a biomarker for drug resistance.

**Methods:**

In total, 133 cases of breast carcinoma treated with neo-adjuvant therapy were collected. Tissue samples from pre-neoadjuvant therapy as well as tissues from post-neo-adjuvant therapy of those cases were collected and stained with immunohistochemistry for TFF3, Bcl2, BAX, cleaved caspase-3, AKT-1, NF kappa B and Ki-67.

**Results:**

There was increased expression of TFF3 in residual invasive carcinoma cells. There was a significant correlation between the expression of TFF3 in breast carcinoma cells and response to neoadjuvant chemotherapy (*p* = 0.0165). There was significant co-expression of TFF3 with AKT1 (*p* = 0.0365), BCl2 (*p* = 0.0152), and NF Kappa-B (*p* = 0.0243) in breast carcinoma cases with residual carcinoma following neoadjuvant therapy which support the role of TFF3 in chemoresistance.

**Conclusion:**

The expression of TFF3 is significantly associated with residual breast carcinoma following neoadjuvant chemotherapy suggesting its expression is associated with increased resistance to chemotherapy. This is supported by its co-expression with antiapoptotic proteins; BCl2, AKT1 and NF Kappa-B in residual breast carcinoma cells and very low proliferating index and apoptotic bodies in residual tumors.

**Electronic supplementary material:**

The online version of this article (10.1186/s12885-019-5316-y) contains supplementary material, which is available to authorized users.

## Background

Cancer is one of the leading causes of death worldwide [1]. In the United Arab Emirates (UAE), it is the third cause of death after cardiovascular diseases and road traffic accidents [2]. Breast carcinoma is the most common malignant neoplasm and the second cause of cancer death in women worldwide [1, 3]. It is the commonest cancer among UAE population and the most common cancer among females. The age specific incidence ratio for females in the UAE is 19.4 per 100,000 populations [2].

Trefoil factor (TFF) peptides are family of small regulatory proteins consisting of three members [4], TFF1 (pS2) [3, 5], originally identified as an estrogen-responsive gene in the MCF-7 human breast cancer cell line, Spasmolytic polypeptide, TFF2 (SP) [3, 6], and intestinal trefoil factor, TFF3 (ITF) [3, 7]. The genes encoding TFF peptides are located in a head to tail orientation, clustered in a 55 kb region on chromosome 21q22.3 [3].

TFF3 peptide has seven conserved cysteine residues, six of which form three intra-chain disulfide bonds that result in a characteristic three-loop structure named trefoil domain or P-domain. This P-domain renders the protein resistant to proteases and acid degradation [7]. TFF3 peptide is normally secreted by intestinal epithelium [3]. It is also detected in many other organs inside the body [3]. TFF3 protein is expressed in normal breast tissue and benign breast disorders. Variable expression of TFF3 has been detected in invasive breast carcinoma; some have shown diminished expression while others have demonstrated increased expression [8, 9]. Examination of the 5′ promoter regions of TFF3 gene has identified putative estrogen and progesterone receptor–DNA binding domains as direct response elements to ovarian hormones that are linked either to endometrial and breast functions or steroid regulation [10]. Resistance of cancer to chemotherapy is an important factor in limiting the effectiveness of chemotherapy. In this study we investigate the association of TFF3 with the development of resistance to chemotherapy.

## Methods

### Case selection

Archival paraffin blocks of invasive breast carcinoma cases before and after neoadjuvant chemotherapy were obtained from the surgical pathology files in the anatomic pathology division at Tawam hospital in Al Ain city, which is the main oncology center in the UAE. In total 133 paraffin blocks of core needle biopsies of invasive breast carcinoma before neoadjuvant chemotherapy and 133 paraffin blocks from mastectomy and lumpectomy specimens of similar cases after neoadjuvant chemotherapy were included in this study. In addition, 16 paraffin blocks of non-neoplastic breast tissue were also be included in this study. The protocol of the present study conformed to the ethical guidelines of the World Medical Association, Declaration of Helsinki, and was approved by Al Ain Medical District Human Research Ethics Committee, (Protocol No. 14/05). Patients or their caregivers signed informed written consent allowing using their anonymous material for research purposes.

### Immunohistochemistry

Five-micrometer sections were prepared and mounted on aminopropyltriethoxysilane (APES) coated slides. After dewaxing with xylene and rehydrating with graded alcohol, slides were placed in a 0.01 M citrate buffer solution (pH = 6.0) and pre-treatment procedures to unmask the antigens was performed in a microwave oven for 10 min. Then, sections were treated with peroxidase block for 20 min followed by protein block for 10 min. Then the sections were stained using streptavidin-biotin immunohistochemical method for TFF3 (mouse monoclonal, clone EP107, Cellmarque, USA), AKT1 (rabbit polyclonal, Santa Cruz biotechnology, USA), NFқB (rabbit polyclonal, Thermo Fisher Scientific, USA), Bcl2 (mouse monoclonal, clone 124, Cellmarque, USA), Bax (rabbit polyclonal, Dako, Agilent, USA), ki-67 (mouse monoclonal, Dako, Agilent, USA), and cleaved casapase-3 (rabbit polyclonal, Asp175, Cell signaling biotechnology, USA). All primary antibodies were applied on sections with appropriate concentration for one hour at room temperature, followed by washing the sections with phosphate buffer saline (PBS) for 15 min in three changes then After conjugation with primary antibodies, sections were incubated with secondary antibody (EnVisionTM Detection System, DAKO, Agilent, USA) for 20 min at room temperature followed by addition of DAB chromogen (EnVisionTM Detection System, DAKO, Agilent, USA) and counter staining done with haematoxylin. Appropriate positive controls were used. For negative control, the primary antibody was not added to sections and the rest of the protocol was carried out as other sections. Positive and negative controls were used in every batch of slides that were stained (not shown in figures).

### Double immunofluorescence staining

Double-Immunofluorescence analysis were done to show co-localization of TFF3 with AKT1, Bcl2 and NFқB in various parts of the tissue sections in different groups. Five-micrometer sections were deparaffinised with xylene and rehydrated with descending concentrations of ethanol. The sections were later incubated with TFF3 (mouse monoclonal, 1:50, Abcam, USA), overnight at room temperature. After 3 times of washing with PBS, sections were incubated with donkey anti-mouse Alexa Fluor 488 (1: 100, Abcam, USA) for 1 h at room temperature. After washing several times, the same sections were incubated overnight at room temperature with a second primary antibodies AKT1 (rabbit polyclonal,1:50, Santa Cruz biotechnology, USA), NFқB (rabbit polyclonal, 1:50, Thermo Fisher Scientific, USA), Bcl2 (rabbit polyclonal, 1:50, Abcam, USA). Sections then were washed in PBS and incubated for 1 h at room temperature with donkey anti rabbit rhodamine (1, 100, Abcam, USA). The sections were then mounted in water-soluble mounting media and viewed with Olympus Fluorescence microscope. Positive and negative controls were used in every batch of slides that were stained (not shown in figures).

### Evaluation of sections

Sections were evaluated by using light microscope with X40 objective. The positive tumor cells were expressed in percentage in each case using ImageJ software (http://rsbweb.nih.gov/ij/). Cases were considered to be positive when more than 1% of malignant cells are positive for particular antibody. The percentage of positive cells were calculated by dividing the number of positive cancer cells per 100 cancer cells. The calculation was repeated in at least 10 random high power fields and the total were divided by 10 to get the mean in each case. The frequency of expression is graded low when expression of particular protein is seen in 2–25% of tumor cells. The frequency of expression is graded intermediate when expression of particular protein is seen in 26–75% of tumor cells. The frequency of expression is graded high when expression of particular protein is seen in > 75% of tumor cells.

### Assessment of response to neoadjuvant therapy

Since our aim in this study is to assess expressions of TFF3 in invasive breast carcinoma before neoadjuvant and after neoadjuvant therapy and whether TFF3 is expressed in residual malignant cells, we have graded the pathological response to neoadjuvant chemotherapy into two groups through reviewing H&E sections on the basis of the parameters used by Chevallier in his study [11].

1. Pathological complete response: Disappearance of all invasive carcinoma or carcinoma in situ in breast with no invasive carcinoma and negative lymph nodes.
2. Pathological incomplete or no response: Presence of invasive carcinoma with stromal fibro-inflammatory changes and reduction of tumor size. There are viability in the extent of response to neoadjuvant therapy ranging from low or no regression with sheets, cords or clusters of malignant cells, to high regression with few residual clusters of malignant cells in one focus or few foci.
  Only the invasive carcinomas and the lymph nodes involvement were graded on the above-mentioned criteria. The presence of lymphovascular emboli and presence of ductal carcinoma in situ were noted separately.

### Evaluation of apoptotic index

The Apoptotic index was calculated by dividing the number of positive cancer cells for anti-casapase-3 per 100 cancer cells. The calculation was repeated in at least 10 random high power fields and the total were divided by 10 to get the apoptotic index.

### Evaluation of proliferative index (ki-67)

The proliferative index was calculated by dividing the number of positive cancer cells for ki-67 per 100 cancer cells. The calculation was repeated in at least 10 random high power fields and the total were divided by 10 to get the proliferative index.

### Statistical analysis

The statistical analysis was computer assisted using SPSS for windows version 20 (SPSS Inc., Chicago, USA). Student’s t-test were used to compare continuous variables. Quantitative variables were analyzed with the chi-squared test and correlations of ordinal variables using the Spearman rank correlation coefficient and Chi-square (Fisher’s exact) test. *P* value < 0.05 were considered significant. Where appropriate numerical data were presented as the mean ± SD.

## Results

### Age distribution of breast carcinoma cases

**Fig. 1:**
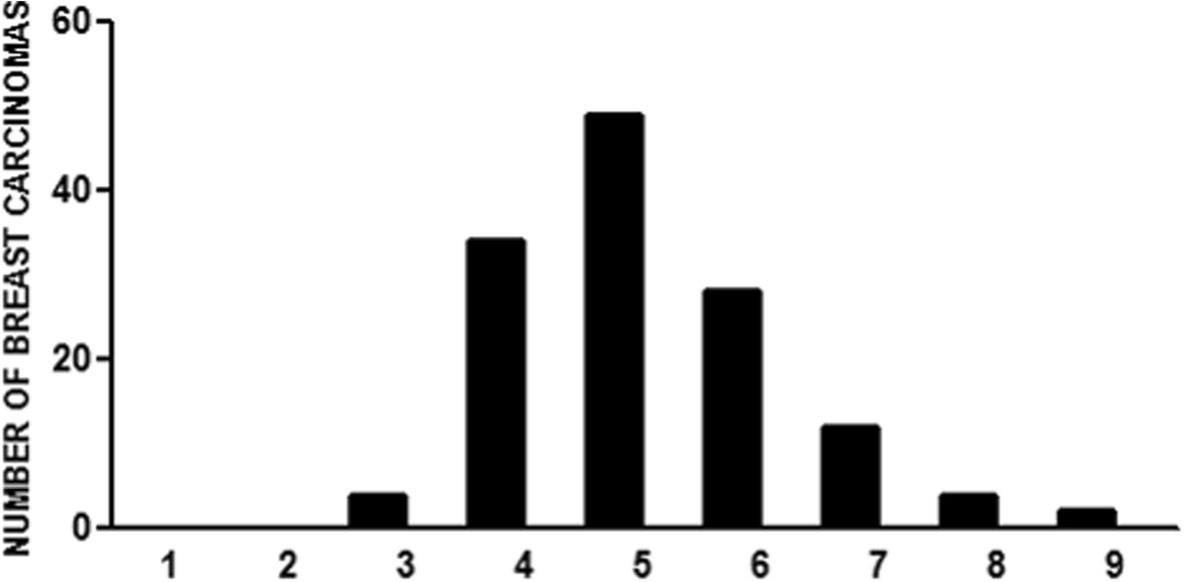
Age distribution of breast carcinoma cases treated with neoadjuvant therapy

In total 133 cases of surgically resectable breast carcinomas treated with neoadjuvant chemotherapy were included in this study. The mean age was 46.8 ± 11.8 years; the median was 45 years and the range 26–85 years. The peak incidence was in the fifth decade (Fig. 1).

### Expression of TFF3 in non-neoplastic tissue

**Fig. 2:**
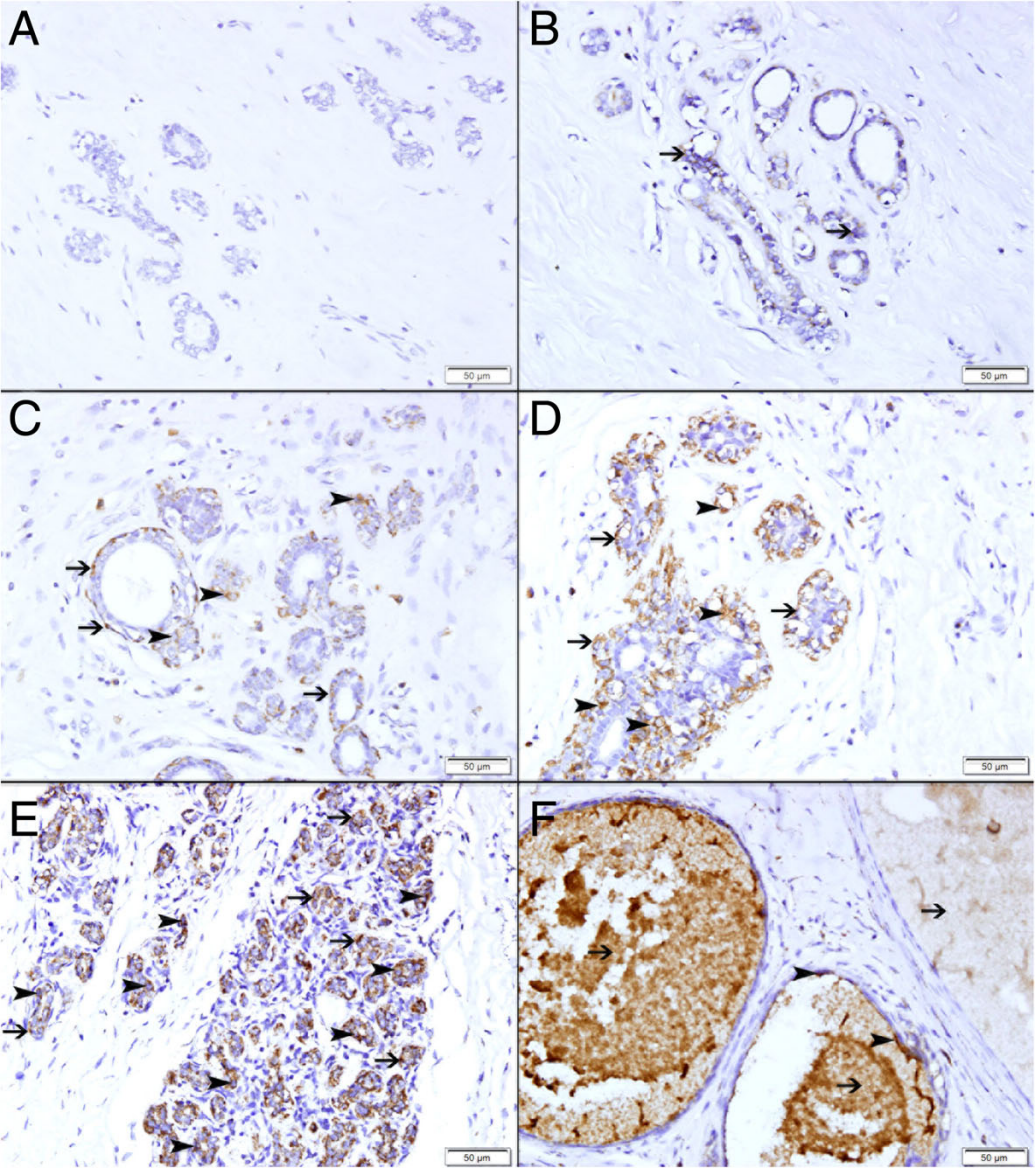
Expression of TFF3 in non-neoplastic breast tissues. **a** Showing no expression of TFF3 in normal breast lobule. **b** Showing low expression of TFF3 by few lobular epithelium (thin arrow). **c** Showing low expression of TFF3 by few lobular epithelium (arrowhead) and myoepithelium (thin arrow). **d** Showing moderate expression of TFF3 by lobular epithelium (arrowhead) and myoepithelium (thin arrow). **e** Showing high expression of TFF3 by lobular epithelium (arrowhead) and myoepithelium (thin arrow). **f** Showing high expression of TFF3 by cells lining the cyst in fibrocystic disease (arrowhead). There is high TFF3 in the fluid content of the cysts (thin arrow)

There was a variable expression of TFF3 by non-neoplastic breast epithelial cells ranging from absence of any expression (Fig. 2a), to low expression (Fig. 2b and c), to intermediate expression (Fig. 2d) and high expression (Fig. 2e). There was also increased expression of TFF3 by epithelial cells forming the lining of cysts in fibrocystic disease of the breast. The fluid of the cyst also shows high TFF3 content (Fig. 2f).

### Expression of TFF3 in ductal carcinoma in situ

**Fig. 3:**
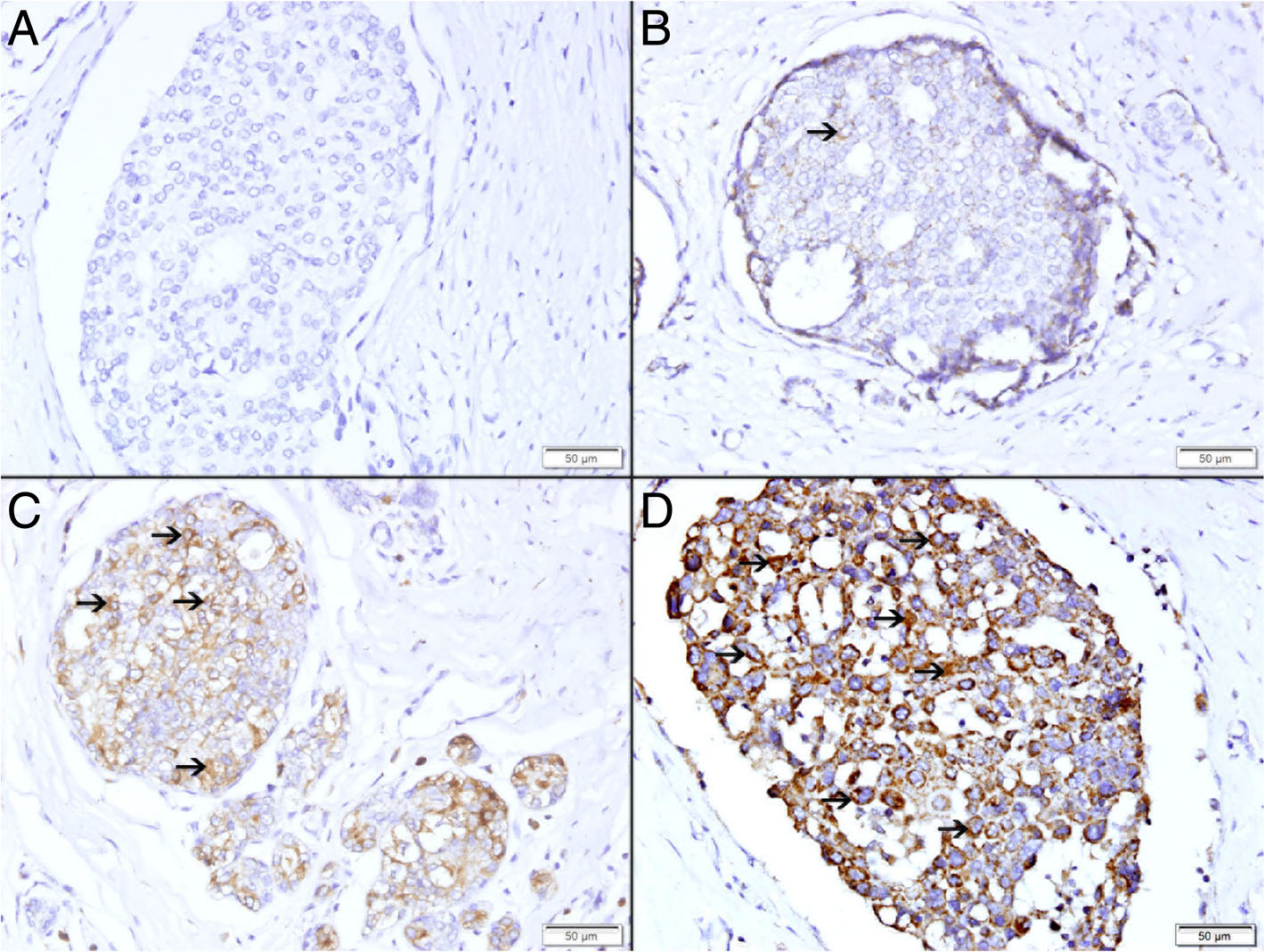
Expression of TFF3 in breast intraductal carcinoma in situ. **a** Showing no expression of TFF3. **b** Showing low cytoplasmic expression of TFF3 (thin arrow). **c** Showing moderate cytoplasmic expression of TFF3 (thin arrow). **d** Showing high cytoplasmic expression of TFF3 (thin arrow)

There was a variable expression of TFF3 by malignant epithelial cells in intraductal carcinoma in situ ranging from absence of any expression (Fig. 3a), to low expression (Fig. 3b), to intermediate expression (Fig. 3c) and high expression (Fig. 3d).

### Expression of TFF3 in invasive breast carcinoma in pre-neoadjuvant core needle biopsies

**Fig. 4:**
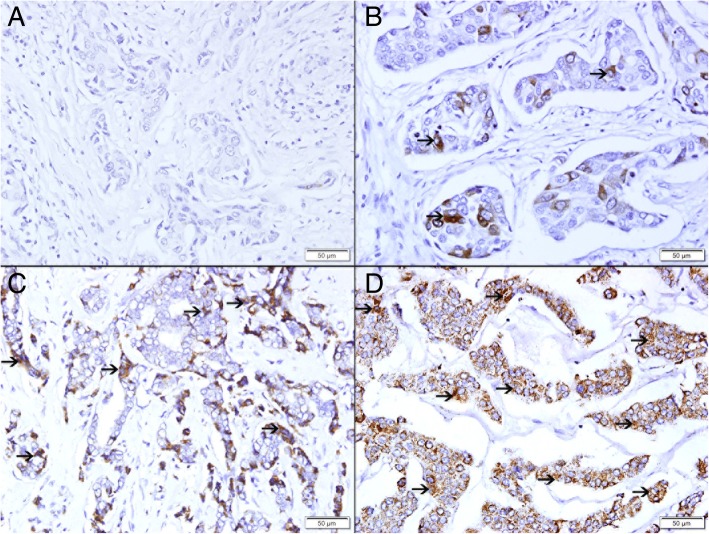
Expression of TFF3 in Pre-neoadjuvant invasive breast carcinoma. **a** Showing no expression of TFF3. **b** Showing low cytoplasmic expression of TFF3 (thin arrow). **c** Showing moderate cytoplasmic expression of TFF3 (thin arrow). **d** Showing high cytoplasmic expression of TFF3 (thin arrow)

There was a variable expression of TFF3 by invasive breast carcinomas. There was no expression of TFF3 in 57 (43%) cases (Fig. 4a). There was variable expression of TFF3 in 76 (57%) of the cases. Low expression of TFF3 is seen in 13 cases (10%) (Fig. 4b), while intermediate (Fig. 4c) and high expression (Fig. 4d) are seen in 32 (24%) and 31 (23%) respectively.

### Response to neoadjuvant therapy

In total 133 cases of breast carcinoma were treated with neoadjuvant chemotherapy.

**Fig. 5:**
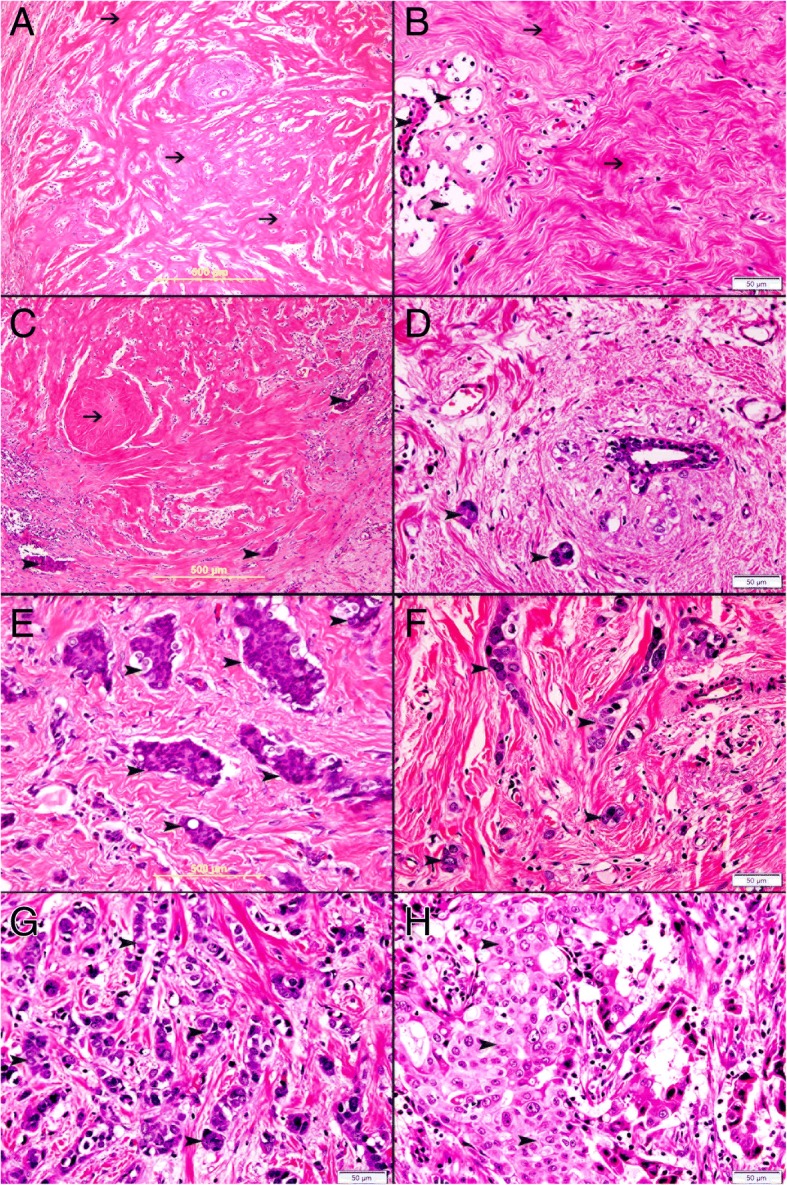
**a** Complete response to neoadjuvant therapy with absence of malignant cells and replacement of malignant cells by hyalinized fibrous tissue (thin arrow). Complete response to neoadjuvant therapy with absence of malignant cells and replacement of malignant cells by hyalinized fibrous tissue (thin arrow) and atrophied breast lobule (arrow head). **c** Incomplete response to neoadjuvant therapy with few residual clusters of invasive ductal carcinoma (arrow head) and replacement of tumor cells by hyalinized fibrous tissue (thin arrow). **d** Incomplete response to neoadjuvant therapy with few residual clusters of invasive ductal carcinoma (arrowhead). **e** Incomplete response to neoadjuvant therapy with clusters of residual invasive ductal carcinoma (arrowhead). **f** Incomplete response to neoadjuvant therapy with residual clusters of invasive ductal carcinoma (arrowhead). **g** Incomplete response to neoadjuvant therapy with many residual clusters of invasive ductal carcinoma (arrowhead). **h** Incomplete response to neoadjuvant therapy with sheets of residual invasive ductal carcinoma (arrowhead)

There was complete response to neoadjuvant chemotherapy with no residual tumor in 33(25%) cases (Fig. 5a and b, while 100 (75%) show incomplete response to neoadjuvant therapy and show residual tumor (Fig. 5c-h).

### Identification of TFF3 in breast cancer cells before and after chemotherapy in pathologic complete response group, pathologic partial response group, and pathologic no response group

**Fig. 6:**
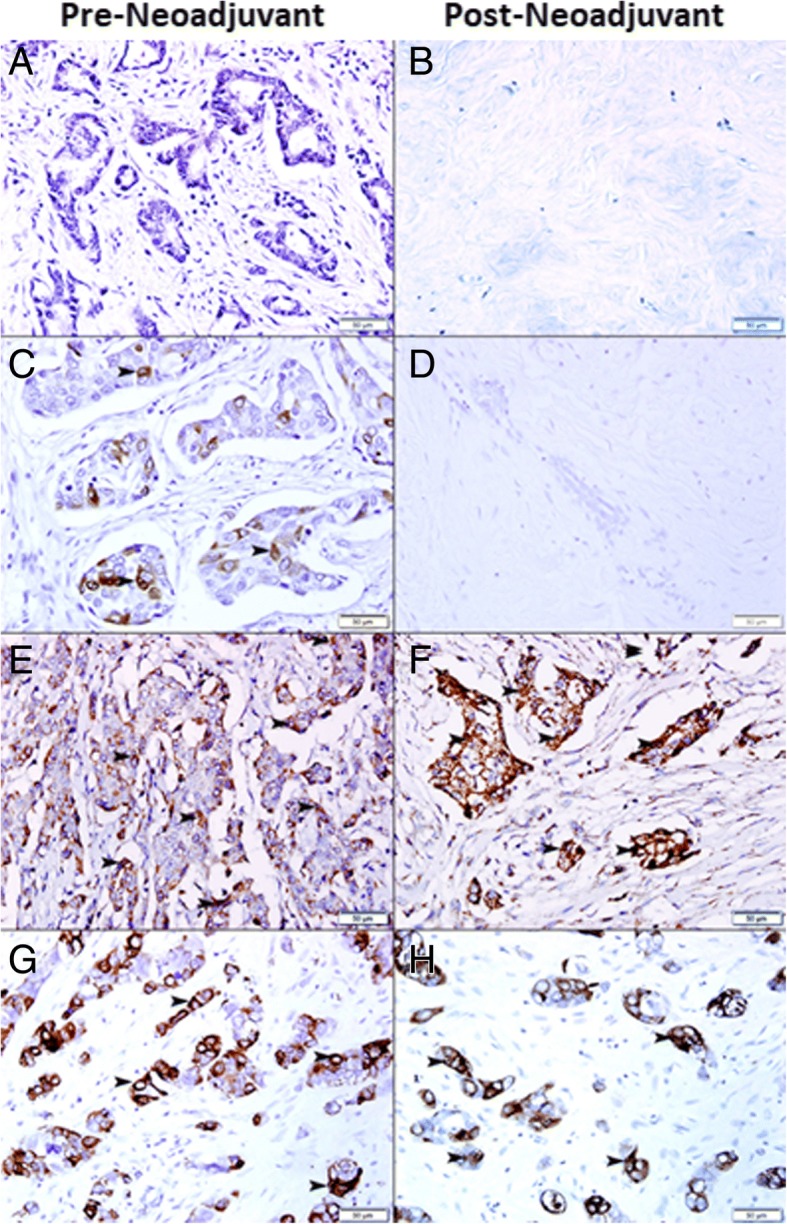
**a** Invasive ductal carcinoma shows no expression of TFF3 in pre-neoadjuvant breast biopsy **b** Complete response to neoadjuvant therapy with no residual tumor. **c** Invasive ductal carcinoma shows mild expression of TFF3 (arrowhead) in pre-neoadjuvant breast biopsy **d** Complete response to neoadjuvant therapy with no residual tumor. **e** Invasive ductal carcinoma shows moderate expression of TFF3 (arrowhead) in pre-neoadjuvant breast biopsy. **f** Incomplete response to neoadjuvant therapy with marked residual malignant cells expressing TFF3 (arrowhead). **g** Invasive ductal carcinoma shows high expression of TFF3 (arrowhead) in pre-neoadjuvant breast biopsy. **h** Incomplete response to neoadjuvant therapy with marked residual malignant cells showing high expression of TFF3 (arrowhead)

In total 27 (85%) of breast carcinoma cases that have complete response to neoadjuvant therapy have shown either no expression (Fig. 6a) or low expression of TFF3 in neoplastic cells at pre-neoadjuvant biopsies (Fig. 6c). Only 6 (15%) cases that have complete response to neoadjuvant therapy have shown moderate TFF3 expression. In total, 22 biopsies show no expression of TFF3 in neoplastic cells at pre-neoadjuvant biopsies while 11 biopsies show low or moderate expression of TFF3 in neoplastic cells at pre-neoadjuvant biopsies.

While 58 (43%) cases of breast carcinoma biopsies with incomplete response or no response to neo-adjuvant therapy have shown moderate (Fig. 6e) to high (Fig. 6g) expression of TFF3 in pre-neoadjuvant biopsies and post neoadjuvant biopsies (Fig. 6f and h).

### Correlation between response to neoadjuvant therapy and expression of TFF3 in breast carcinoma cells following resection

**Fig. 7:**
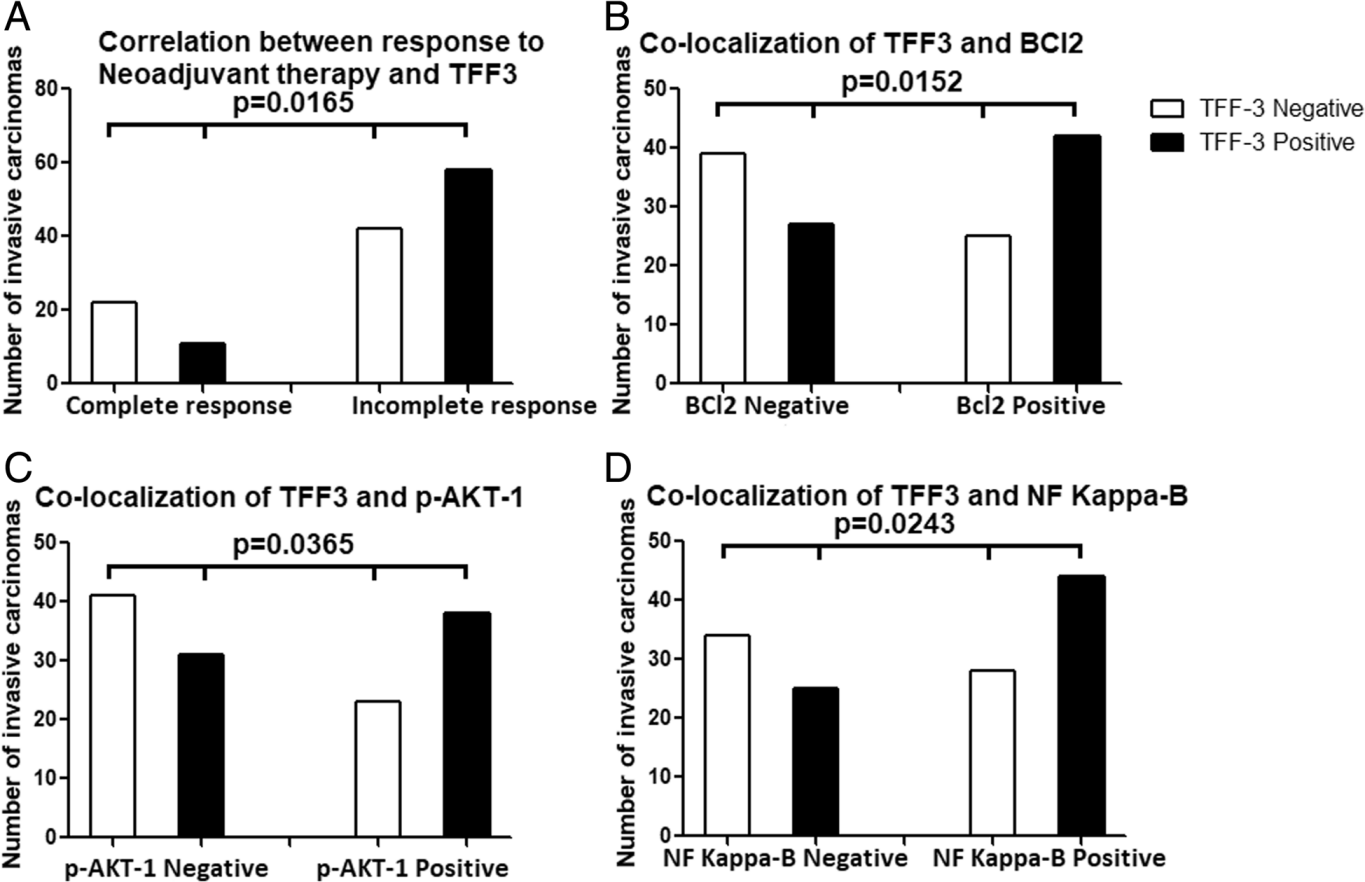
**a** Correlation between response to neoadjuvant therapy and expression of TFF3. **b** Correlation between TFF3 and BCl2 expression in breast carcinoma cases treated with neoadjuvant therapy. **c** Correlation between TFF3 and AKT1 expression in breast carcinoma cases treated with neoadjuvant therapy. **d** Correlation between TFF3 and NF Kappa-B expression in breast carcinoma cases treated with neoadjuvant therapy

There was a significant correlation between the expression of TFF3 in breast carcinoma cells and response to chemotherapy (*p* = 0.0165) as 58% (58) of residual carcinomas express TFF3 and 66% (22) of cases with complete response did not express TFF3 in pre-neoadjuvant biopsies (Fig. 7a) (Additional file 1: Table S1). In addition, the cases with residual carcinoma that express TFF3 in resection specimens, they also show expression of TFF3 in pre-neoadjuvant biopsies, since the expression of TFF3 is similar in pre-neoadjuvant core needle biopsies and resection specimens with residual carcinoma. This indicates that TFF3 expression is associated with resistance to chemotherapy as it is highly expressed in residual carcinoma.

### Co-localization of TFF3 in relation with Bcl2, p-AKT1, and NF kappa-B expression in residual tumors

**Fig. 8:**
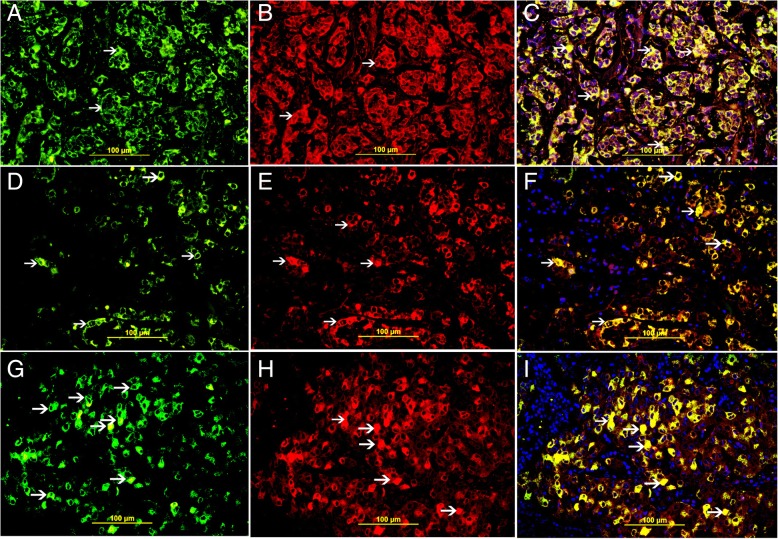
**a** Cytoplasmic expression of TFF3 in residual carcinoma cells (thin arrow). **b** Cytoplasmic expression of BCl2in residual carcinoma cells (thin arrow). **c** Cytoplasmic Co-localization of TFF3 and BCl2 (thin arrow). **d** Cytoplasmic expression of TFF3 in residual carcinoma cells (thin arrow). **e** Cytoplasmic expression of p-AKT1 in residual carcinoma cells (thin arrow). **f** Cytoplasmic Co-localization of TFF3 and p-AKT1 (thin arrow). **g** Cytoplasmic expression of TFF3 in residual carcinoma cells (thin arrow). **h** Cytoplasmic expression of NF Kappa-B in residual carcinoma cells (thin arrow). C. Cytoplasmic Co-localization of TFF3 and NF Kappa-B (thin arrow)

Co-localization of TFF3 with Bcl2, p-AKT-1 and NF kappa-B was done by double labeling immunofluorescent technique and it show high expression of Bcl2, p-AKT-1 and NF Kappa-B in neoplastic cells of residual tumors following neoadjuvant therapy which also show high expression of TFF3 (Fig. 8).

### Correlation of expression of TFF3 and BCl2 in residual breast carcinoma cases treated with neoadjuvant therapy

There was significant co-expression of TFF3 and BCl2 in breast carcinoma cases treated with neoadjuvant therapy (*p* = 0.0152) suggesting anti-apoptotic role of TFF3 (Fig. 7b and 8c), (Additional file 1: Table S2).

### Correlation of expression of TFF3 and AKT1 in residual breast carcinoma cases treated with neoadjuvant therapy

There was significant co-expression of TFF3 and AKT1 in breast carcinoma cases treated with neoadjuvant therapy (*p* = 0.0365) which supports the anti-apoptotic role of TFF3 (Fig. 7c and 8f), (Additional file 1: Table S3).

### Correlation of expression of TFF3 and NF kappa-B in residual breast carcinoma cases treated with neoadjuvant therapy

There was significant co-expression of TFF3 and NF Kappa-B in breast carcinoma cases treated with neoadjuvant therapy (*p* = 0.0243). NF Kappa-B is a growth signaling pathway and when it is stimulated it can also inhibit apoptosis which also supports the anti-apoptotic role of TFF3 (Fig. 7d and 8i), (Additional file 1: Table S4).

### Effect of proliferative index in pre-neoadjuvant biopsies on the outcome in post neoadjuvant therapy

There was no significant relation between proliferative index of breast carcinoma in pre-neoadjuvant biopsies and post-neoadjuvant outcome (*P* = 0.4778).

### Effect of apoptotic index in pre-neoadjuvant biopsies on the outcome in post neoadjuvant therapy

There was no significant relation between apoptotic index of breast carcinoma in pre-neoadjuvant biopsies and post-neoadjuvant outcome (*P* = 0.869).

### Correlation between post neoadjuvant residual carcinoma and proliferative index

**Fig. 9:**
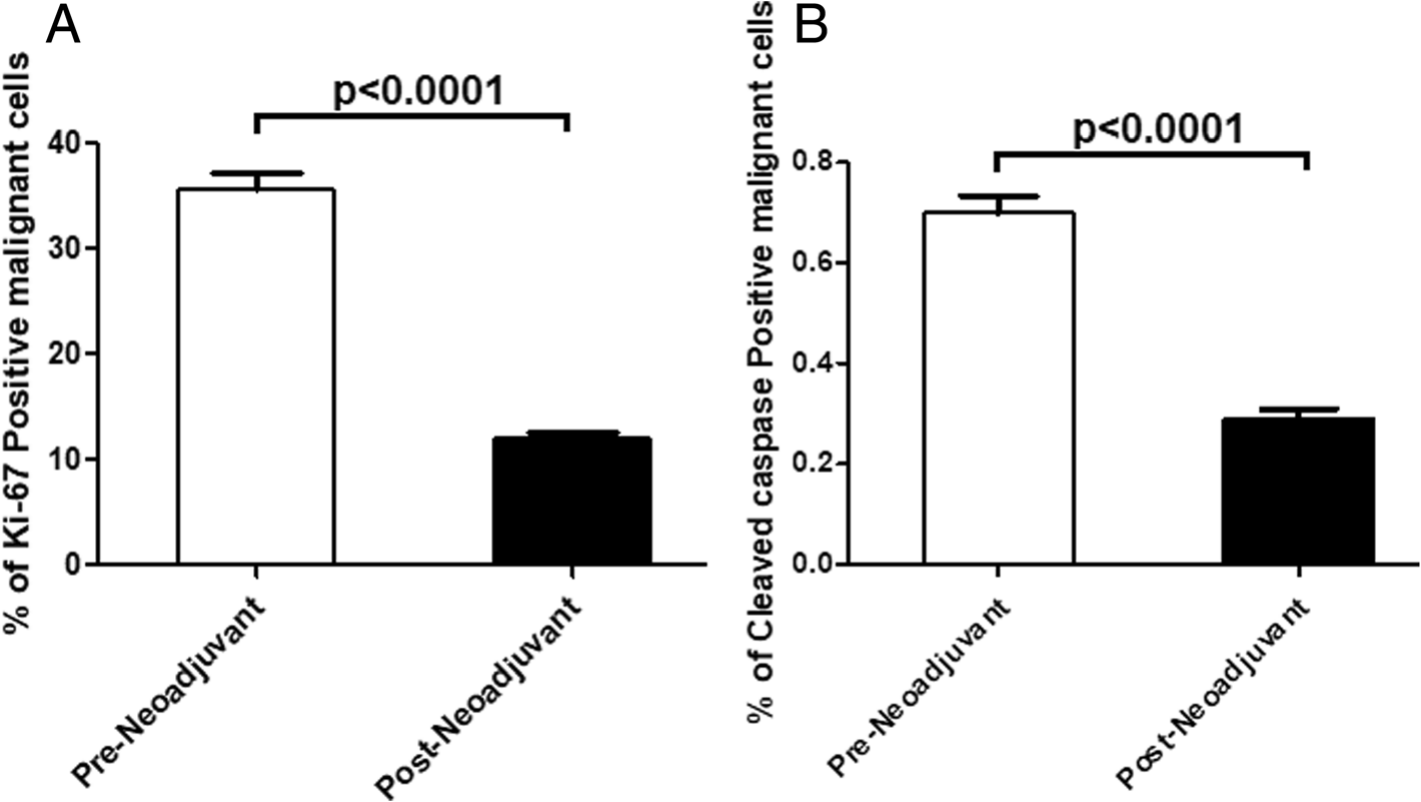
**a** Proliferative index in pre-neoadjuvant tumors and post neoadjuvant residual tumors. **b** Apoptotic index in pre-neoadjuvant tumors and post neoadjuvant residual tumors

There was a significant decrease in proliferative index in post-neoadjuvant breast carcinoma cells when compared with pre-neoadjuvant proliferative activity (*P* < 0.0001) (Fig. 9a). In many cases of residual carcinoma there was no expression of ki-67 by malignant cells indicating loss proliferative activity by these cells.

### Correlation between post neoadjuvant residual carcinoma and apoptotic index

There was a significant decrease in apoptotic index in post-neoadjuvant breast carcinoma cells when compared with pre-neoadjuvant proliferative activity (*P* < 0.0001) (Fig. 9b). In many cases of residual carcinoma there was no expression of cleaved caspase 3 by malignant cells indicating increased antiapoptotic activity in these cells.

### Correlation between TFF3 expression, apoptotic bodies and cell proliferation in breast carcinoma following neoadjuvant therapy

**Fig. 10:**
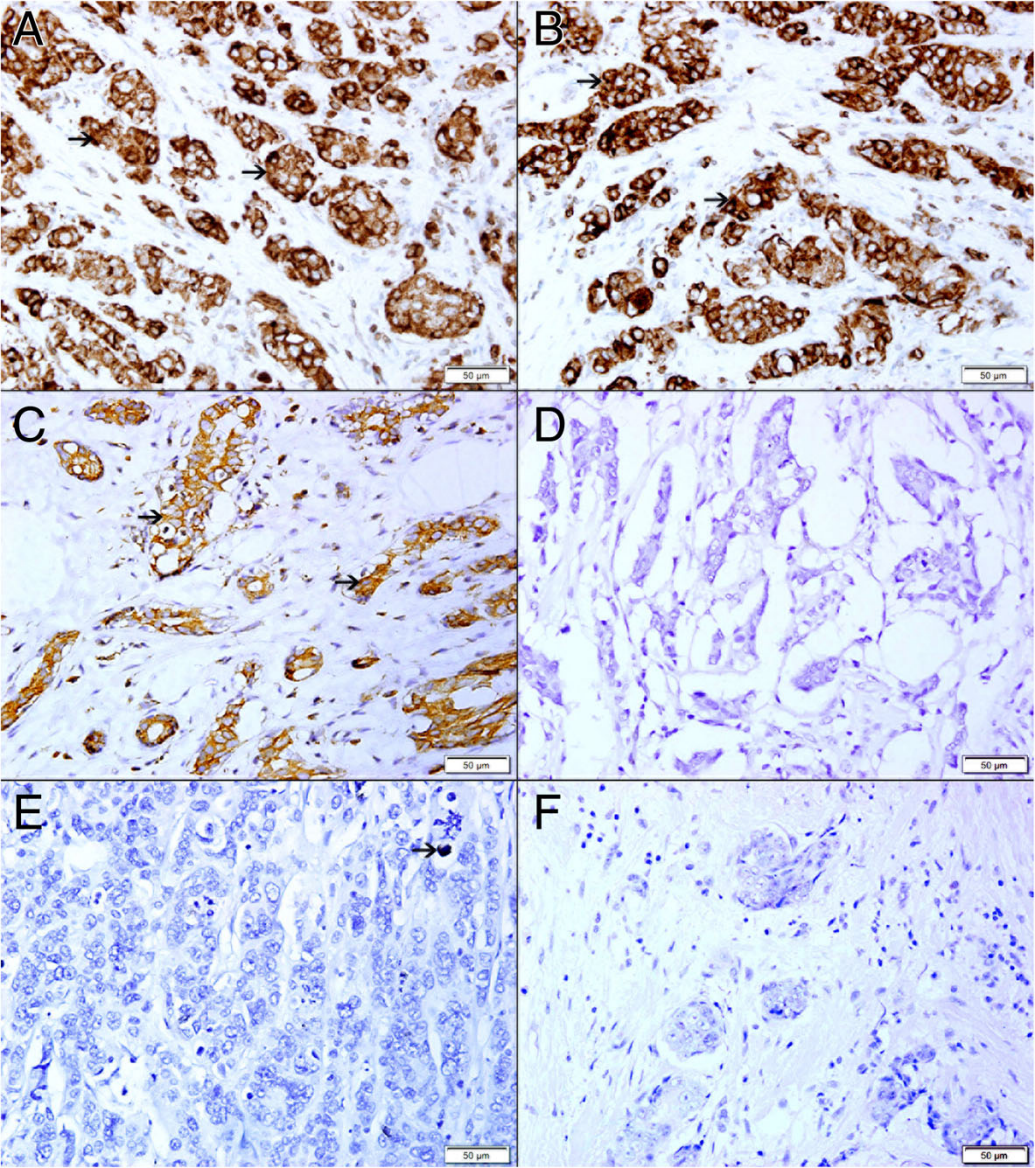
Residual tumors proliferative and apoptotic activity. **a** High expression of TFF3 in residual neoplastic cells (arrow). **b** High expression of Bcl2 in residual neoplastic cells (arrow). **c** High expression of AKT-1 in residual neoplastic cells (arrow). **d** No expression of BAX in residual neoplastic cells. **e** Very low expression of cleaved caspase-3 in residual neoplastic cells (arrow). No expression of KI-67 in residual neoplastic cells

There was very low or no expression of ki67 protein (Fig. 10f) in residual tumor as compared to high expression of TFF3 in residual tumor cells (Fig. 10a) suggesting that residual tumor cells have a low proliferating activity, this is supported by the high expression of Bcl2 (Fig. 10b) and p-AKT-1 (Fig. 10c) by residual tumor cells.

There is also a very low apoptotic activity in residual tumor cells supported by very low expression of Bax (Fig. 10d) and cleaved caspase 3 (Fig. 10e) by residual tumor cells and high expression Bcl2 and AKT-1 by residual tumor cells (Fig. 10b and c).

## Discussion

Breast carcinoma is the most common cancer among females worldwide. Neoadjuvant chemotherapy is one of the modalities of treating breast carcinomas which ultimately followed by surgery. Neoadjuvant chemotherapy proved to be beneficial in down staging cancers, allowing for less extensive afterward surgery, improved cosmetic outcomes, and reduced postoperative complications such as lymphedema [12]. Resistance to chemotherapy is one of the challenges that can affect the outcome of neoadjuvant chemotherapy. Many factors are involved in the development of resistance to chemotherapy. Chemoresistance can be due to intrinsic factors where cancer cells are resistant before their exposure to chemotherapy or to an acquired resistance developed during chemotherapy [13–15]. Resistance to apoptosis is an important factor in the development of resistance to chemotherapy [14].

We have shown a significant correlation between the expression of TFF3 in breast carcinoma cells and response to neoadjuvant chemotherapy. We have shown the majority of residual carcinomas in incomplete pathological response group are expressing high level of TFF3 in pre-neoadjuvant and post neoadjuvant specimens, while the majority of cases with complete pathological response group have no or low expression of TFF3 in pre-neoadjuvant biopsies. This suggest that high expression of TFF3 by malignant cells is associated with resistance to neoadjuvant chemotherapy [16]. We have also shown co-localization of TFF3 with anti-apoptotic protein in residual breast carcinoma. We have also shown significant reduction in proliferative and apoptotic activities in cancer cells of residual tumors in conjunction with high expression of TFF3 in the majority of residual tumors. These findings outline a possible anti-apoptotic role of TFF3 in invasive breast carcinoma.

Taupin et al. have shown that TFF3-expressing HT29 cell line is resistant to induced apoptosis and exogenous TFF3 can protect HCT116 and IEC-6 cell lines from apoptosis [17]. They have also shown that TFF3 knock-out mice have increased intestinal apoptosis [17]. We also have identified a significant reduction in apoptotic bodies in residual tumor together with high expression of TFF3 suggesting a possible antiapoptotic role of TFF3 in incomplete pathological response group. It is noteworthy to mention here that many of the residual clusters of invasive breast carcinoma that show high expression of TFF3 have no apoptotic cells.

It has been shown that TFF3 mediates resistance to apoptosis through activation of EGFR and PI3-kinase in colonic HT29 cells [18]. Durual et al. have shown that PI3K promotes the expression of TFF3 and MUC2 and that the PI3-K/Akt pathway may play a pivotal role in intestinal goblet cell differentiation. They have also shown specific PI3K inhibitor can inhibit TFF3 and MUC2 expression in intestinal epithelium [19–22]. The anti-apoptotic effect required intact TFF3 dimmer and phosphorylation of the EGFR [23]. On the same track we also have shown a significant co-localization of TFF3 and p- AKT-1 in breast carcinoma cells in residual tumors suggesting a possible association between TFF3 and AKT-1 in incomplete pathological response group.

TFF3 can also block apoptosis in a p53-dependent manner after challenge with the topoisomerase inhibitor etoposide in human colonic cancer cells HCT116 and in the non-transformed rat intestinal epithelial cell line IEC-6 [17]. Moreover, Taupin  et al. have demonstrated that both endogenous and exogenous TFF3 may prevent p53-dependent and p53-independent apoptosis [17]. Casado et al. have shown overexpression of TFF3 and Bcl2 in colonic carcinoma following neoadjuvant therapy suggesting that TFF3 might induce Bcl2, block apoptosis and prevent colonic cancer cell death following neoadjuvant therapy [24]. This is compatible with our finding of co-localization of TFF3 and BCl2 in residual invasive carcinoma in incomplete pathological response group.

We also have identified a significant co-localization between TFF3 and NF Kappa B in breast carcinoma cells in residual tumor suggesting a possible role in the development of incomplete pathological response. This is supported by Zhu et al. study  who have also shown that TFF3, which activates NF-қB in a transient event, up regulates Twist protein in intestinal epithelial cells and protects epithelial cell from death by apoptosis. This effect of TFF3 is mediated by endogenous ERK activity [25–27]. Chen et al. have shown that TFF3-treated cells are resistant to apoptosis, through the transcription factor NF-kB pathway, a key mediator of TFF-dependent cell survival [28].

These studies [17–28] supports our findings and point towards antiapoptotic role of TFF3 in residual breast carcinoma. The increased expression of TFF3 in breast carcinoma will promote their survival and make them resistance to neoadjuvant therapy.

## Conclusion

The expression of TFF3 is significantly associated with residual breast carcinoma following neoadjuvant chemotherapy suggesting its expression is associated with increased resistance to chemotherapy. This is supported by its co-expression with antiapoptotic proteins; BCl2, AKT1 and NF Kappa-B in residual breast carcinoma cells and very low proliferating index and apoptotic bodies in residual tumors.

## Additional file

Additional file 1: **Table S1.** Correlation between TFF3 expression and response to neoadjuvant chemotherapy. **Table S2.** Correlation between TFF3 expression and BCl2 expression in residual invasive carcinomas following neoadjuvant chemotherapy. **Table S3.** Correlation between TFF3 expression and p-AKT-1 expression in residual invasive carcinomas following neoadjuvant chemotherapy. **Table S4.** Correlation between TFF3 expression and NF Kappa-B expression in residual invasive carcinomas following neoadjuvant chemotherapy. (DOCX 14 kb)

## Abbreviations

AKT-1: RAC-alpha serine/threonine-protein kinase
BCl2: B cell lymphoma-2
EGFR: Epidermal growth factor receptor
ERK: Extracellular signal-regulated kinases
MUC2: Mucin 2
NF kappa B: Nuclear factor kappa B
TFF3: Trefoil factor 3
