# Supplementary material for: Trefoil factors peptide-3 is associated with residual invasive breast carcinoma following neoadjuvant chemotherapy
Source: BMC Cancer. 2019 Feb 11;19:135. doi: 10.1186/s12885-019-5316-y (PMC6371459; doi:10.1186/s12885-019-5316-y)
Supplement: Supplementary file 1 — Table S1. Correlation between TFF3 expression and response to neoadjuvant chemotherapy. Table S2. Correlation between TFF3 expression and BCl2 expression in residual invasive carcinomas following neoadjuvant chemotherapy. Table S3. Correlation between TFF3 expression and p-AKT-1 expression in residual invasive carcinomas following neoadjuvant chemotherapy. Table S4. Correlation between TFF3 expression and NF Kappa-B expression in residual invasive carcinomas following neoadjuvant chemotherapy. (DOCX 14 kb) [file 12885_2019_5316_MOESM1_ESM.docx]

**Additional file 1**

**Table S1** Correlation between TFF3 expression and response to neoadjuvant chemotherapy.

| **Pathologic Response to Chemotherapy** | **TFF3** | | **Total** |
| --- | --- | --- | --- |
|  | **Negative** | **Positive** |  |
| **Complete** | **22** | **11** | **33** |
| **Incomplete** | **42** | **58** | **100** |
| **Total** | **64** | **69** | **133** |

**Table S2** Correlation between TFF3 expression and BCl2 expression in residual invasive carcinomas following neoadjuvant chemotherapy.

| **BCl2** | **TFF3** | | **Total** |
| --- | --- | --- | --- |
|  | **Negative** | **Positive** |  |
| **Negative** | **39** | **27** | **66** |
| **Positive** | **25** | **42** | **67** |
| **Total** | **64** | **69** | **133** |

**Table S3** Correlation between TFF3 expression and p-AKT-1 expression in residual invasive carcinomas following neoadjuvant chemotherapy.

| **p-AKT1** | **TFF3** | | **Total** |
| --- | --- | --- | --- |
|  | **Negative** | **Positive** |  |
| **Negative** | **41** | **31** | **72** |
| **Positive** | **23** | **38** | **61** |
| **Total** | **64** | **69** | **133** |

**Table S4** Correlation between TFF3 expression and NF Kappa-B expression in residual invasive carcinomas following neoadjuvant chemotherapy.

| **NF Kappa-B** | **TFF3** | | **Total** |
| --- | --- | --- | --- |
|  | **Negative** | **Positive** |  |
| **Negative** | **36** | **25** | **61** |
| **Positive** | **28** | **44** | **72** |
| **Total** | **64** | **69** | **133** |
